# Increased Expression of the Tail-Anchored Membrane Protein SLMAP in Adipose Tissue from Type 2 *Tally Ho* Diabetic Mice

**DOI:** 10.1155/2011/421982

**Published:** 2011-07-13

**Authors:** Xiaoliang Chen, Hong Ding

**Affiliations:** ^1^Second People Hospital of Hangzhou, No. 1 Wenzhou Road, Hangzhou 310015, China; ^2^Department of Pharmacology, Weill Cornell Medical College in Qatar, P.O. Box 24144, Doha, Qatar

## Abstract

The tail-anchored membrane protein, sarcolemmal membrane associated protein (SLMAP) is encoded to a single gene that maps to the chromosome 3p14 region and has also been reported in certain diabetic populations. Our previous studies with db/db mice shown that a deregulation of SLMAP expression plays an important role in type 2 diabetes. Male *Tally Ho* mice were bred to present with either normoglycemia (NG) or hyperglycemia (HG). Abdominal adipose tissue from male *Tally Ho* mice of the HG group was found to have a significantly lower expression of the membrane associated glucose transporter-4 (GLUT-4) and higher expression of SLMAP compared to tissue from NG mice. There were 3 isoforms expressed in the abdominal adipose tissue, but only 45 kDa isoform of SLMAP was associated with the GLUT-4 revealed by immunoprecipitation data. Knock down studies using SLMAP siRNA with adipocytes resulted in a significant reduction in SLMAP and a decrease in glucose uptake. Thus, SLMAP may be an important regulator of glucose uptake or involved in GLUT-4 fusion/translocation into the plasma membrane of mouse abdominal adipose tissue and changes in SLMAP expression are linked to hyperglycemia and diabetes.

## 1. Introduction

The etiology of type 2 diabetes is complex and involves both genetic predisposition as well as environmental factors, notably lifestyle and dietary influences, and has been closely linked to the obesity epidemic [1, 2]. In the study of type 2 diabetes murine models have become an important tool for determining the molecular basis for disease progression and tissue dysfunction. In the *db/db* mouse type 2 diabetes is linked to a dysfunctional leptin receptor, and the mouse is hyperphagic, massively obese with marked hyperglycemia and has been widely used for the study of type 2 diabetes [3, 4]. The *Tally Ho* mouse is a polygenic mouse model of type 2 diabetes that is susceptible to obesity [5]. It has been argued that the *Tally Ho* mouse is a good model for human diabetes as genetic analysis of this mouse by Jackson Laboratories suggested that diabetes is linked to a major susceptibility locus on chromosome (Chr) 19 and interactions with additional sites on Chr 16 [1] and with an obesity gene ob chromosome 6 [6]. 

Syntaxins are docking molecules that share the structure of a very short COOH terminus and a long cytoplasmic NH_2_-terminal region encompassing two coiled-coil domains [7]. The SLMAPs, like several tail anchoring proteins, such as syntaxin and soluble N-ethylmaleimide-sensitive factor attachment protein receptor (SNARE), are involved in the docking and membrane fusion process via protein-protein interactions [8, 9]. Studies have shown that syntaxin and SNARE-complex proteins are required in translocation of glucose transporters GLUT-1 and GLUT-4 [10, 11]. 

We have reported previously that endothelial dysfunction in *db/db* mice is associated with a significant upregulation of the expression of the tail-anchored membrane protein, SLMAP [12]. Furthermore, treatment with the PPAR*γ* agonist, COOH, reversed the endothelial dysfunction in *db/db* mice and corrected the aberrant expression of vascular SLMAP [12]. These data suggested that changes in the expression of the SLMAP gene might contribute to the development of diabetes. The *Tally Ho*, like the *db/db* mouse, also shows vascular and endothelial dysfunction [13, 14]. In the current study we have further pursued the link between SLMAP expression and type 2 diabetes by utilizing the polygenic type 2 diabetic *Tally Ho* mouse model. *Tally Ho *mice also have a higher adiposity index compared to control C57BL/6J mice [5], and our goal in the present study was therefore to study SLMAP expression in *Tally Ho* mice and determine whether there was a link with alterations in adipocyte function, namely, GLUT-4 and glucose regulation.

## 2. Materials and Methods

### 2.1. Animals and Tissue Handling

Breeding pairs of *Tally Ho* mice were purchased from Jackson Laboratories (Bar Harbor, ME, USA) and bred at Monash University, VIC, Australia. In accordance with a protocol approved by RMIT University Animal Ethics Committee, 24-week-old male mice were sacrificed by cervical dislocation. It was originally reported that the *Tally Ho* mice exhibit mild obesity, hyperinsulinemia, hyperlipidemia, and male-specific hyperglycemia and the animal colony was established through a selective breeding process based on the hyperglycemic phenotypes by choosing mating males showing high plasma glucose levels and then inbreeding from hyperglycemic males and apparently normal females [5]. In this study, we have selected the mice for the study groups according to plasma glucose levels. The mice were separated into two groups according to glucose levels with those below 12 mmol/L placed in the normoglycemic group (NG) and above 24 mmol/L into the diabetic hyperglycemic group (HG). The abdominal fat was dissected out for Western blot and real-time PCR studies. 

### 2.2. Real-Time PCR

Total RNA was extracted from abdominal fat using an RNeasy Mini Kit with on-column DNase treatment (QIAGEN), and first-strand cDNA was subsequently synthesized using a Superscript RT Kit (QIAGEN). Real-time PCR primers were designed as previously described [12] with the addition that the expression levels of GLUT-4 were also determined. The following primer sequences for GLUT-4 were used: F: 5′ CCTCCTGCTTGGCTTCTTC 3′; R: 5′ GTTTCACCTCCTGCTCTAAAAG 3′. As previously described [12] the sequence homology of the isoforms for SLMAP prevents the primer designed for real-time PCR from distinguishing between the different SLMAP isoforms, and thus the RT-PCR data reflects changes in cumulative SLMAP message levels for the tissues and animals studied. 

### 2.3. Antibodies, Immunoblotting, and Immunoprecipitation

Adipose tissue was isolated from *Tally Ho* mice and homogenized in RIPA lysis buffer. Polyclonal antibodies against SLMAP were characterized previously [15], and antibodies against GLUT-4 and actin were purchased from Abcam and Santa Cruz Biotechnology Inc. Protein samples (50 ug per lane) were separated by SDS-PAGE in the presence of dithiothreitol and transferred to nitrocellulose membranes. The membranes were blocked with 5% milk in Tris-buffered saline and incubated with primary antibody for 1 h. The nitrocellulose membranes were washed three times with Tris-buffered saline-Tween (0.05%) solution and incubated with horseradish peroxidase-conjugated second antibody for 1 h. The reaction was visualized by chemiluminescence. The Western blot data was quantified using Chemidoc densitometry (Biorad). 

### 2.4. Coimmunoprecipitation

Aliquots of lysate (300 *μ*g protein) were incubated with 1 *μ*g of normal rabbit IgG, and the lysates were precleared by the addition of 20 *μ*L of protein A/G-Sepharose (Santa Cruz Biosciences) for 1 h. After centrifugation, the resulting supernatant was incubated with 1 *μ*g of anti-SLMAP antibody or anti-GLUT-4 antibody overnight at 4°C with gentle rocking. Immune complexes were absorbed on protein G-Sepharose and washed 4 times with PBS. Finally immunoblotting was performed as described above. 

### 2.5. Subcellular Fractionation

Subcellular protein fractions from cytosolic and membrane components were extracted using Qproteome cell compartment Kit (QIAGEN). Equal protein content (20 ug per lane) of fractions was analyzed by SDS-PAGE and immunoblotting.

### 2.6. Adipocyte Cell Isolation and Culture

Adipose tissue (5-6 g) from *Tally Ho* mice was finely minced and gently shaked for 60 min at 37°C in 50 mL Krebs-Ringer bicarbonate (KRB) buffer (in mmol/L: NaCl 118, MgSO_4_ 1.2, CaCl_2_ 1.3, NaHCO_3_ 2.5, pH adjusted to 7.4 at 37°C) containing 1 mmol/L pyruvate, 1% bovine serum albumin, and 0.1% collagenase. The cell suspension was then filtered through a nylon mesh (400 *μ*mol/L) and centrifuged at 100 g for 1 min at room temperature. The supernatant was harvested and washed three times with 50 mL of KRB buffer containing 1 mmol/L pyruvate and 1% BSA. Adipocytes were cultured in Dulbecco's modified Eagle's medium (DMEM), 10% fetal bovine serum, 100 U/mL penicillin, and 100 *μ*g/mL streptomycin. Culture media contained either 10 mM or 30 mM glucose. 10 mM and 30 mM glucose were chosen for this protocol as plasma glucose levels were comparable to those reported for the db/db leptin receptor mutant diabetic mouse as well as the *Tally Ho* mice in this study [16]. Cell cultures were maintained in an incubator at 37°C, with saturating humidity and an atmosphere of 5% carbon dioxide to 95% air.

### 2.7. 2-Deoxy[^3^H]Glucose Uptake Measurement

Cells were seeded into 6-well plates. After reaching 80% confluence, the cells were washed and incubated with 800 uL of DMEM (0.1% FBS) containing 1 uCi of 2-Deoxy-D-Glucose (Perkin Elmer) and 10 uM of nonradiolabeled glucose (Sigma) for 30 min. The cells were treated with SLMAP or negative control siRNAs. After incubation the cells were washed with 1 mL ice-cold PBS 3x then lysed with 1 mL NaOH (0.3 M). 800 uL of the lysate was then used for glucose uptake assay, and the remaining lysates were used for protein assay.

### 2.8. SLMAP Knockdowns

 SLMAP and negative control siRNAs were purchased from QIAGEN based on sequences of mouse SLMAP (Accession no. NM_032008). The single-stranded siRNA sequences targeting SLMAP were 5′-CGUGAUGUGCAUGAUUUAAd(-TT)-3′ and 5′-AGAUGAAGAUAGACUCUUAd(TT)-3′, and sequence for negative control was ACGUGACACGUUCGGAGAAd(TT). After reaching 80% confluence cultured in DMEM with 10% FCS and antibiotics as described earlier, cells were transfected with 5 nM siRNA using HiPerfect Transfection Reagent in a 6-well plate according to the manufacturer's instructions. After 72 h of incubation, SLMAP expression and glucose uptake were studied.

### 2.9. Data Analysis

In all experiments *n* equals the number of animals used in the protocol. Statistical significance of difference between means of different groups was performed using Student's *t*-test or one-way ANOVA. A value of *P* < 0.05 was considered statistically significant.

## 3. Results

*Tally Ho* mice were assigned into two groups according to their blood glucose level. The normoglycemic group (NG) mice had blood glucose level lower than 12 mmol/L, while the hyperglycemic group (HG) mice had blood glucose higher than 24 mmol/L. The mean blood glucose levels in NG and HG groups were 9.9 ± 1.1 and 27.3 ± 3.4, respectively (*P* < 0.05). The body weights and serum insulin level were comparable between the NG nondiabetic *Tally Ho* mice and the HG diabetic *Tally Ho* mice (35.0 ± 2.8 g versus 33.3 ± 1.2 g and 0.9 ± 0.3 ug/L versus 0.6 ± 0.2 ug/L) (*P* > 0.05).

**Figure 1:**
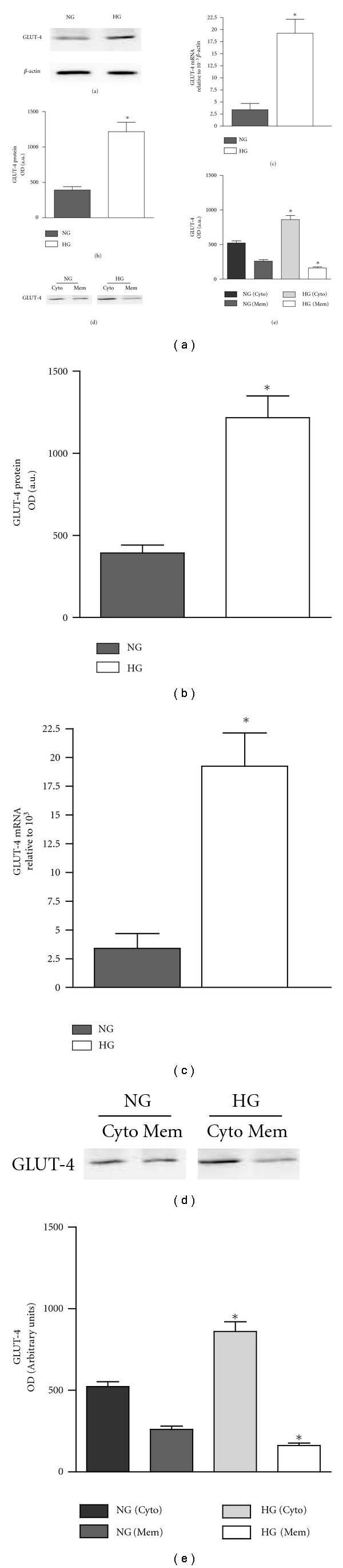
Expression of GLUT-4 in adipose tissue in *Tally Ho *mice. Adipose tissue from both NG and HG *Tally Ho* mice was isolated and analyzed by immunoblotting with anti-GLUT-4 (a). The expression of the GLUT-4 was quantified by densitometry and is shown as bar graph (b). Real-time PCR for GLUT-4 message normalized to *β*-actin from both NG and HG *Tally Ho* mice (c). Subcellular membrane fractions Cyto (cytosolic fraction) and Mem (membrane fraction) from adipose tissue in both NG and HG *Tally Ho* mice were analyzed by immunoblotting with anti-GLUT-4 (d). The expression of GLUT-4 from cytosolic and membrane fractions was quantified by densitometry and is shown as bar graph (e). The data are expressed as mean ± SEM. 4 to 6 mice were included in each group. *Significantly different (*P* < 0.05) *n* = 4.

GLUT-4 protein expression in adipose tissue from HG mice was significantly higher than that in NG mice (Figure 1(a)). There was a 3.3 ± 0.5-fold increase of GLUT-4 expression in adipose tissue from HG *Tally Ho* mice compared to that from NG mice (Figure 1(b)) (*P* < 0.05). Real-time PCR showed that the GLUT-4 mRNA transcript levels of adipose tissue from HG *Tally Ho* mice were 6.4 ± 1.2-fold higher compared with those from NG *Tally Ho* mice (Figure 1(c)) (*P* < 0.05). However, compartmentalization studies showed that increased total GLUT-4 expression from adipose tissue of HG mice contributed to significantly increased GLUT-4 expression in the cytosolic fraction while membrane-bound GLUT-4 was significantly decreased compared to that from adipose tissue of NG mice (Figures 1(d) and 1(e)) (*P* < 0.05).

**Figure 2:**
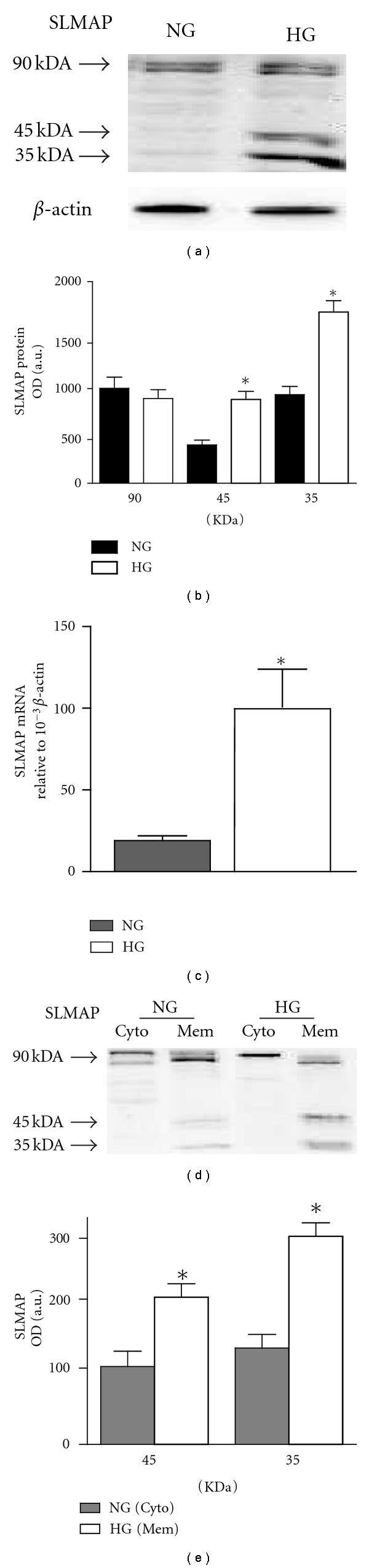
Expression of SLMAP isoforms in adipose tissue in *Tally Ho *mice. Adipose tissue was isolated and analyzed by immunoblotting with anti-SLMAP (a). Anti-SLMAP immunoreactive proteins are depicted by arrows, and the positions of migration of the molecular weight standards are shown as numbers in (kilodaltons). The expressions of the 90, 45, and 35 kDa SLMAP from adipose tissue in both NG and HG *Tally Ho* mice were quantified by densitometry and are shown as bar graph (b). Real-time PCR for SLMAP message normalized to *β*-actin from both NG and HG *Tally Ho* mice (c). Subcellular membrane fractions Cyto (cytosolic fraction) and Mem (membrane fraction) from adipose tissue in both NG and HG *Tally Ho* mice were analyzed by immunoblotting with anti-SLMAP (d). The expression of the 35 and 45 kDa isoforms of SLMAP from membrane fraction was quantified by densitometry and shown as bar graph (d,e). The data are expressed as mean ± SEM. 4 to 6 mice were included in each group. *Significantly different (*P* < 0.05) *n* = 4.

Specific antibodies raised against SLMAP fusion protein were used to study SLMAP expression in the *Tally Ho* mouse adipose tissue.  Figure 2(a) shows that the anti-SLMAP antibody recognized three polypeptides of approximately 90, 45, and 35 kDa in adipose tissue from both NG and HG *Tally Ho* mice. The SLMAP expression profile in the adipose tissue revealed that the levels of expression of the 45 kDa and the 35 kDa SLMAP protein in adipose tissue from HG mice were markedly higher than those from NG mice (Figure 2(a)). The 90 KDa isoform was not significantly different between NG and HG *Tally Ho* mice. The Western blot data of the SLMAP expression were quantified, and it was evident that HG *Tally Ho* mice exhibited a 1.9 ± 0.1-fold increase in expression of the 45 kDa and a 2.0 ± 0.2-fold increase in the 35 kDa SLMAP isoforms compared to NG *Tally Ho* mice (Figure 2(b)) (*P* < 0.05). Thus, these results indicate that the expressions of the 45 kDa and the 35 kDa SLMAP isoforms were specifically enhanced in adipose tissue from HG *Tally Ho* mice.

SLMAP transcript levels of adipose tissue from NG and HG *Tally Ho* mice were also examined by real-time PCR. mRNA levels of SLMAP were 6.1 ± 0.9-fold higher in the adipose tissue (Figure 2(c)) (*P* < 0.05) from HG *Tally Ho* mice compared with that from NG *Tally Ho* mice. 

Compartmentalization studies showed that the 90 kDa isoform of SLMAP was predominant in the cytosolic fraction while the 35 and 45 kDa isoforms were mainly in the membrane fraction (Figure 2(d)). There were a 2.0 ± 0.2 fold increase and a 2.3 ± 0.2 fold increase of membrane-bound 35 and 45 kDa isoforms, respectively, in adipose tissue from HG *Tally Ho* mice compared with that from NG *Tally Ho* mice (Figure 2(e)) (*P* < 0.05). 

**Figure 3:**
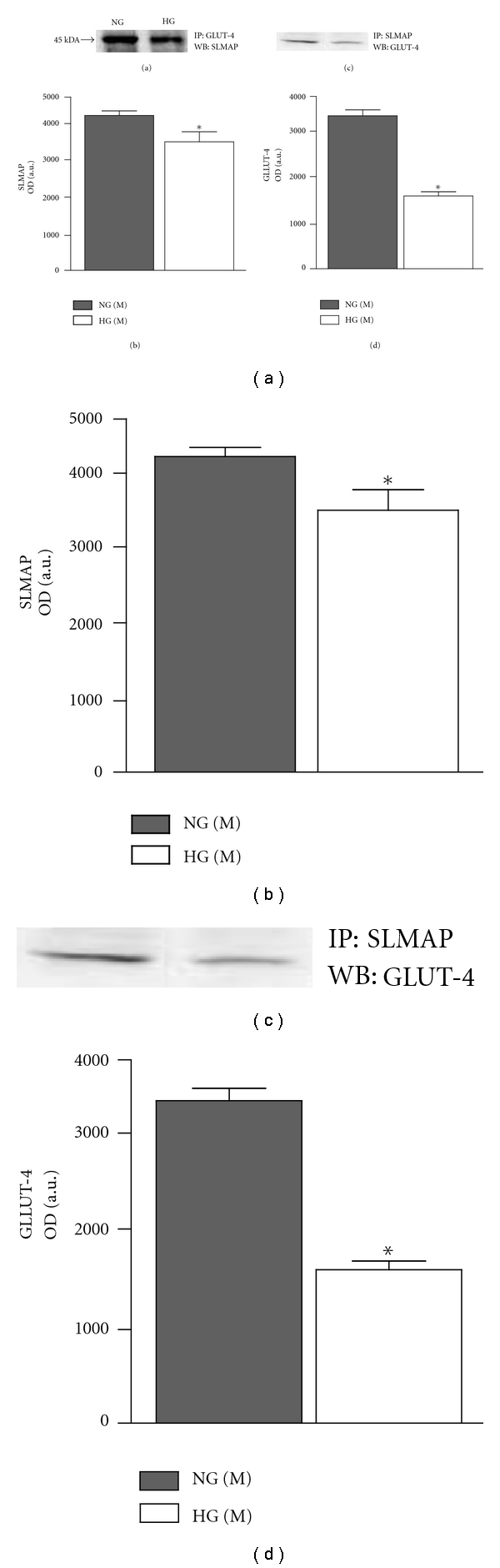
Immunoprecipitation for SLMAP and GLUT-4 in subcellular membrane fractions from adipcytes. Subcellular membrane fractions were immunoprecipitated with GLUT-4 and SLMAP antibody and immunoblotted by SLMAP and GLUT-4 antibody, respectively (a,c). The protein expression levels were quantified by densitometry and are shown as bar graph (b,d). *Significantly different (*P* < 0.05) *n* = 4.

Subcellular membrane and cytosolic fractions were immunoprecipitated with GLUT-4 antibody and immunoblotted by SLMAP antibody. Even though both 45 and 35 kDa SLMAP isoforms were expressed in the membrane fraction, only the 45 kDa isoform was detected by Western blot. The association of GLUT-4 and SLMAP was significantly decreased to 68.5% ± 6.3 in HG mice compared to that in NG mice (Figures 3(a) and 3(b)) (*P* < 0.05). The association of GLUT-4 and SLMAP was also studied by immunoprecipitation with SLMAP antibody and immunoblotted by GLUT-4 antibody in the subcellular membrane fractions. The association of GLUT-4 and SLMAP was decreased to 38.6 ± 2.0% compared to that observed in NG mice (Figures 3(c) and 3(d)) (*P* < 0.05). However, no band could be detected using cytosolic fraction and immunoprecipitation (data not shown).

**Figure 4:**
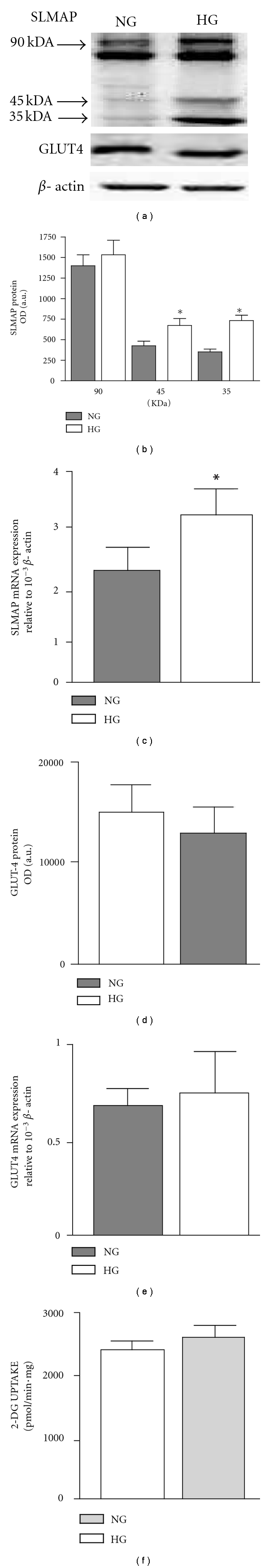
SLMAP expression in adipocytes. Representative gels for protein expression of SLMAP, GLUT-4, and *β*-actin in adipocytes cultured with 10 mM (NG) and 30 mM glucose (HG) for 3 days by Western blot method (a); data quantified by densitometry and are shown as bar graph for SLMAP (b) and GLUT-4 (d). Real-time PCR for SLMAP (c) and GLUT-4 (e) message normalized to *β*-actin from both adipocytes cultured with NG and HG for 3 days. Glucose uptake was measured in both adipocytes treated with NG and HG using radiolabeled 2-Deoxy-D-Glucose and data expressed in the bar graphs (f). *Significantly different (*P* < 0.05) *n* = 4.

Adipocytes isolated from NG *Tally Ho* mice were cultured in 10 mM (NG) or 30 mM (HG) glucose culture media for 3 days. The SLMAP expression profile revealed that the 90 kDa SLMAP was the predominant isoform and was not significantly different between the adipocytes in NG media and HG media. The levels of expression of the 45 kDa and the 35 kDa SLMAP protein in adipocytes cultured in HG media were markedly higher than those cultured in NG media (Figure 4(a)). The Western blot data for SLMAP expression were quantified, and there were a 1.6 ± 0.1-fold increase in expression of the 45 kDa and a 2.1 ± 0.2-fold increase in the 35 kDa SLMAP isoforms in adipocytes cultured in HG compared to expression levels in NG (Figure 4(b)) (*P* < 0.05). mRNA levels of SLMAP were 1.5 ± 0.2-fold higher in the adipocytes cultured in HG compared to those in NG (Figure 4(c)) (*P* < 0.05). There were no significant difference for mRNA and protein expression levels of GLUT4 between adipocytes cultured in HG and NG media (Figures 4(a), 4(d), and 4(e)) (*P* > 0.05). There was no significant difference between glucose uptake in adipocytes treated with NG compared to uptake in those treated with HG (Figure  4(f)) (*P* > 0.05).

**Figure 5:**
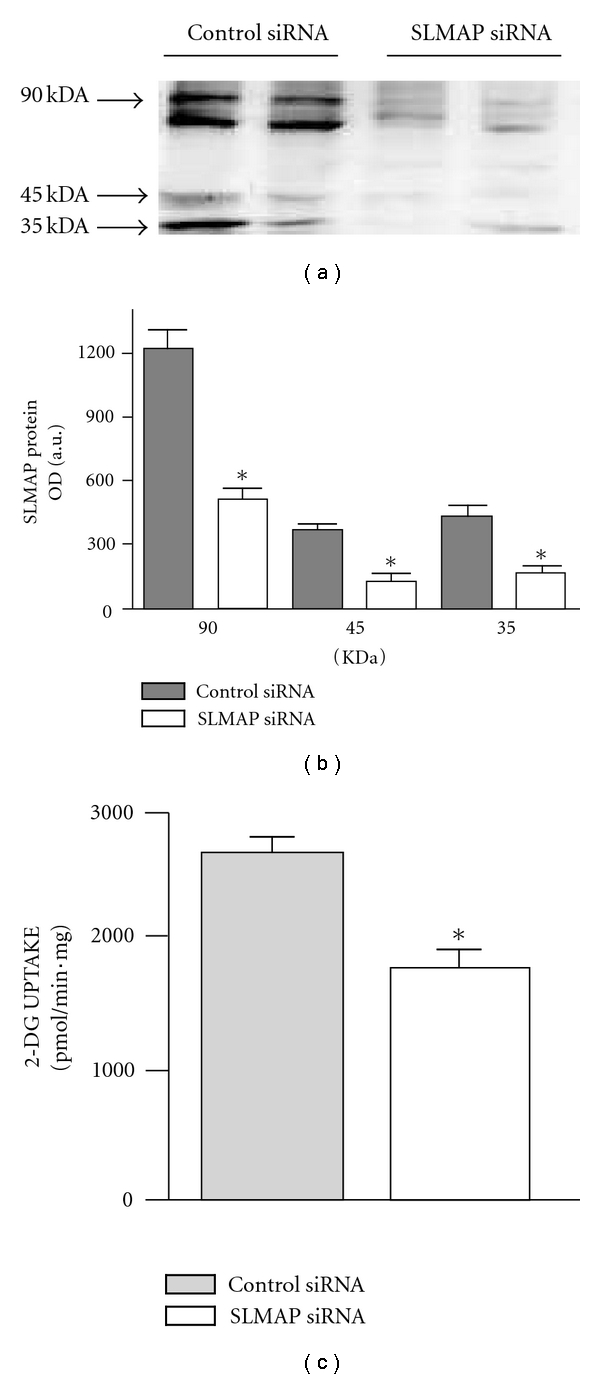
knockdown SLMAP using siRNA in adipocytes. Representative gel for adipocytes treated with SLMAP siRNA to knockdown SLMAP and those treated with a scrambled siRNA as negative control (a). The total expression of SLMAP was quantified by densitometry and shown as bar graph (b). Glucose uptake was measured in both adipocytes treated with SLMAP siRNA and negative control using radiolabeled 2-Deoxy-D-Glucose and data expressed in the bar graphs (c). *Significantly different (*P* < 0.05) *n* = 4.

After adipocytes were treated with SLMAP siRNA for 3 days, total SLMAP protein expression for all isoforms was significantly decreased to 38.7% ± 4.6 when compared to SLMAP protein levels in adipocytes treated with negative control siRNA (Figures 5(a) and 5(b)) (*P* < 0.05). There was no significant difference in GLUT4 expression in adipocytes following treatment with SLMAP or negative control siRNAs (data not shown). Glucose uptake in adipocytes treated with SLMAP siRNA was significantly decreased to 61.7% ± 7.1 compared to uptake in adipocytes treated with negative control siRNA (Figure 5(c)) (*P* < 0.05).

## 4. Discussion

The new findings from the present study are the following. Firstly, in abdominal adipocytes from the *Tally Ho *mouse, a polygenic model of type 2 diabetes, there was an upregulation of the 45 and 35 KDa isoforms of the tail-anchored membrane protein SLMAP in HG mice. The expression of these two isoforms in adipocytes isolated from NG mice is also upregulated after culturing in HG medium. Both isoforms were predominantly expressed in the membrane fraction. Secondly, although both the mRNA and protein expression levels of GLUT-4 were significantly elevated in adipose tissue from HG* Tally Ho *mice, membrane-bound GLUT-4 was decreased compared to NG *Tally Ho *mice. Thirdly, immunoprecipitation data indicated that there was an association of GLUT-4 with the 45 kDa membrane-bound isoform of SLMAP thus suggesting a possible role for SLMAP in the regulation of glucose transport. In addition, even though both the association of GLUT-4 with SLMAP and membrane-bound GLUT-4 were decreased in abdominal adipocytes from HG compared to those from NG, there was no difference between glucose uptake in adipocytes treated with NG and HG suggesting a possible compensatory role of up regulated SLMAP. Fifth and finally, when expression of SLMAP was knocked down in adipocytes with siRNA, glucose uptake was significantly decreased. Collectively these data suggest that SLMAP is involved in GLUT-4 regulation in adipose tissue and abnormalities in the regulation of SLMAP expression in adipose tissue from diabetic *Tally Ho* mice may be linked to alterations in glucose transport and the etiology of diabetes in this animal. 

The data presented in the current study is in accordance with data from the leptin-receptor-deficient (*db/db*) mice which also exhibits the typical features of type 2 diabetes with hyperglycemia, dyslipidemia, and hyperinsulinemia [17–22]. We have previously reported that the vasculature was dysfunctional in *db/db* mice and this functional impairment was closely associated with a marked dysregulation in expression of the tail-anchored membrane protein SLMAP in the *db/db* microvasculature [12]. The reduced endothelial function in the *db/db* vasculature was accompanied by a 2.7-fold increase in the expression of a 35 kDa SLMAP isoform. It is noteworthy that SLMAP upregulation was unaltered in the myocardium from these animals suggesting that changes in SLMAP regulation are not simply a secondary response to the diabetic state, that is, hyperglycemia. 

Of interest is that the vasculature of the *Tally Ho* mouse shows similar endothelial and vascular dysfunction as that reported for the *db/db* mouse [13, 14, 23]. An upregulation of the 45 and 35 KDa isoforms of the tail-anchored membrane protein SLMAP is evident in both adipocytes and vasculature but not in skeletal muscle and heart in HG *Tally Ho* mice (Ding, unpublished data). The present data linking dysregulation of SLMAP expression to GLUT-4 in adipocytes from *Tally Ho* mice reinforces our conclusion that a dysregulation of SLMAP expression is linked, in a tissue-specific manner, to diabetes.

SLMAPs define a new family of tail-anchored membrane proteins that exhibit tissue-specific expression with the highest level of expression seen in cardiac and smooth muscle [15, 24]. SNAREs are tail-anchored membrane proteins known to regulate GLUT-4 translocation in diabetic models [25]. SNAREs play an important role in regulating insulin-stimulated translocation of GLUT-4 from an intracellular compartment to plasma membrane and glucose uptake in adipose tissue. Our preliminary data demonstrated that SLMAP protein levels can be modulated by insulin-resistant states and by agents that increase insulin sensitivity [12]. Maier et al. reported that GLUT-4 levels in skeletal muscle from Zucker diabetic rats were similar to those in lean control rats; however, SNAREs were elevated in skeletal muscle from Zucker diabetic rats [26]. Restoration of normoglycemia and normoinsulinemia in Zucker rats with rosiglitazone normalized SNARE protein levels; however, an elevation of SNAREs was not seen in the streptozotocin-induced diabetic rat leading to the conclusion that changes in SNARE expression are linked to hyperinsulinemia rather than hyperglycemia. In *db/db* mice treatment with the PPAR*γ* agonist COOH also normalized metabolic dysfunction and SLMAP expression in the microvasculature [12]. In this study, we have selected the mice for the study groups according to plasma glucose levels. Although, in the mice we studied, there is no significant difference in the insulin levels between NG and HG groups, this is consistent with other studies that show only mild hyperinsulinemia or reduced insulin secretion of *Tally Ho* mice. For instance, Sung et al. have measured plasma insulin concentrations during oral glucose tolerance test and found that the plasma insulin concentrations were lower in *Tally Ho* mice than in C57BL/6 mice [27]. The *Tally Ho* mice investigated in the current study although hyperglycemic did not have elevated insulin levels thus suggesting that expression of SLMAP is affected by hyperglycemia and not insulin.

Cells cotransfected with constructs encoding Myc-SLMAP and GFP-SLMAP variants confirmed that SLMAPs were homodimerized [28]. The ability of SLMAPs to reside in different membrane systems and to homodimerize may allow this molecule to serve a role in membrane organization. Changes in vesicle transport, which is critical for the movement and recycling of proteins such as GLUT-4, to and from the cell surface may lead to changes in glucose handling.

Numerous SNARE-related proteins have particular cellular and organellar specificity, and each protein demonstrates apparent specificity for a single kind of vesicle or target membrane to ensure vesicle-to-target specificity [29]. Tail-anchored proteins show selectivity to their targets by posttranslational modification such as N-glycosylation. Insertion of a C-terminal N-glycosylation sequence into the tail-anchored protein cytochrome b5 resulted in localization exclusively to mitochondrial outer membrane instead of endoplasmic reticulum [30]. The alternative exons XI, XII, and XIII of the SLMAP gene were predicted to introduce posttranslational modifications sites including phosphorylation for PKC, as well as N-glycosylation and N-myristoylation [9]. Further studies are required to test if alterations of SLMAP phosphorylation and glycosylation in hyperglycemia may cause malfunction and mislocalization of SLMAP that leads to alteration of GLUT-4 and regulation of glucose transportation.

In adipocytes from HG *Tally Ho* mice, the expression of SLMAP was significantly increased; however, the association between SLMAP and GLUT-4 was decreased in hyperglycemic conditions. Glucose uptake in adipocytes treated with NG or HG remained unchanged. We thus hypothesized that SLMAP expression was up regulated to compensate for a decreased role of SLMAP in GLUT-4 transfer/fusion to the plasma membrane. That glucose uptake of adipocytes isolated from *Tally Ho* mice was significantly decreased after siRNA knockdown of SLMAP expression supports a role for SLMAP in the regulation of glucose transporters. 

In conclusion, in adipose tissue both the tail-anchored membrane protein SLMAP and GLUT-4 expression are up regulated in HG* Tally Ho *mice. However, the association between GLUT-4 and SLMAP was decreased in the diabetic mice when compared to the NG controls, perhaps reflecting that changes in SLMAP expression play a compensatory role in diabetes. The association between GLUT-4 and SLMAP in adipocytes and the ability of siRNA knockdown of SLMAP to reduce both SLMAP expression and glucose uptake suggests a possible regulatory role of SLMAP in GLUT-4 fusion and translocation to the plasma membrane and in the regulation of glucose uptake. The altered association of GLUT-4 and SLMAP in the hyperglycemic state suggests that alterations in the regulation of SLMAP expression might play an important role in the development of diabetes.
